# Editorial: Deep learning in crop diseases and insect pests

**DOI:** 10.3389/fpls.2023.1145458

**Published:** 2023-02-13

**Authors:** Peng Chen, Rujing Wang, Po Yang

**Affiliations:** ^1^ National Engineering Research Center for Agro-Ecological Big Data Analysis & Application, Information Materials and Intelligent Sensing Laboratory of Anhui Province, Institutes of Physical Science & Information Technology and School of Internet, Anhui University, Hefei, Anhui, China; ^2^ Institute of Intelligent Machines, Hefei Institutes of Physical Science, Chinese Academy of Sciences, Hefei, Anhui, China; ^3^ Department of Computer Science, Sheffield University, Sheffield, United Kingdom

**Keywords:** deep learning, BigData analysis, crop diseases and insect pests, feature representation, plant sensor

Many deep learning methods have been developed and successfully applied in the field of crop pests and diseases detection. In crop pest identification, deep learning methods can achieve good feature representation from large datasets based on various linear and nonlinear deep learning transformations and then discover the relationship in complex data based on specific supervised and unsupervised learning. However, with the in-depth study of plant diseases and pest infestations, deep learning technology also has limitations. The current agricultural infrastructure is the first limitation that is not yet sufficient to fully support the application of deep learning in the agricultural field. This requires a large number of computational resources and has a high time complexity caused by too many network parameters. The second reason is the lack of a large amount of labeled data and the subjectivity of manually labeled data in the agricultural domain. Moreover, it is difficult to obtain large-scale images of plant diseases and pests in real fields, and it is impossible to acquire images of multiple diseases and pests in one area.

At the same time, the detection of plant diseases and pests is limited by the complex background, illumination conditions, overlapping and occlusion of leaves, and similar color of foreground and background. In addition, there are other problems in the application of deep learning methods for plant pest detection, such as gradient disappearance and gradient explosion in the training process of the network, and overfitting of the network model. The most important problem is that most current deep learning networks are still considered as black-box models. Misidentification by a network for crop pest and disease detection can lead to disastrous results. For example, misidentification of the severity of crop damage can lead to the overuse of pesticides, which in turn can lead to soil contamination, environmental damage, and other vicious cycles.

In order to improve the identification and detection of crop pests and diseases, we propose this Research Topic “*Deep Learning in Crop Diseases and Insect Pests*” for the development of novel deep learning-based methods in crop pests and diseases detection. The Research Topic contains 16 original research articles based on detection of eight plant diseases, including those on grapes, strawberry, potato, pear, tomato etc., and detection of eight plant pests, focusing on tomato pest, wheat spike, etc. Eight papers developed different deep learning-based methods in this Research Topic for detection of crop diseases. Here five papers focused on specific crop, such as potato, grape, tomato, and strawberry. Yuan et al. presented an improved DeepLab v3+ deep learning network for the segmentation of grapevine leaf black rot spots to evaluate grape disease grade. The DeepLab v3+network uses ResNet101 as the backbone network, a channel attention module inserted into the residual module, and a feature fusion branch based on a feature pyramid network to fuse feature maps of different levels. Plant Village and from an orchard field test sets were used for testing the segmentation performance of the method. Li et al. proposed an integrated framework to realize the segmentation and detection of potato foliage diseases in complex backgrounds, combining instance segmentation model of Mask R-CNN to segment potato leaves in complex backgrounds, classification models of VGG16, ResNet50 and InceptionV3 to classify potato leaves, and semantic segmentation models of UNet, PSPNet, and DeepLabV3+ to divide potato leaves. It is important to detect the devastating diseases of potato early blight and late blight that affect potato planting and production. Albahli and Nawaz presentd a robust approach, namely the DenseNet-77-based CornerNet model, for the localization and classification of the tomato plant leaf abnormalities in the complex incidences of light variation, color, brightness changes, and the occurrence of blurring and noise on the 10 classes of tomato leaf images. You et al. proposed a strawberry disease detection scheme with unknown diseases, where the known strawberry diseases are detected with deep metric learning (DML)-based classifiers along with the unknown diseases that have certain symptoms. The DML-based post-filtering stage contains two different types of classifiers: softmax classifiers that are only for known diseases and the K-nearest neighbor (K-NN) classifier for both known and unknown diseases. The proposed scheme can be applied to identify disease-like symptoms caused by real known and unknown diseases or disorders for any kind of plant. Jiang et al. proposed two different but related deep learning techniques for the detection of unknown plant diseases; Open Set Recognition (OSR) and Out-of-Distribution (OoD) detection. OSR is premature to be applied in finegrained recognition tasks without outlier exposure that a certain part of OoD data (also called known unknowns) are prepared for training, where OoD detection requires intentionally prepared outlier data during training.

Moreover, two papers focused on disease detection for public datasets of crop diseases. Xia et al. devoted to plant disease identification and subtype discovery through a deep-embedding image-clustering strategy, Weighted Distance Metric, and the t-stochastic neighbor embedding algorithm (WDM-tSNE), which has been tested on public datasets of images, such as MNIST database (Modified National Institute of Standards and Technology database), PlantVillage, Aphanomyces Root Rot Image Dataset. Xu et al. proposed a transfer learning strategy with a vision transformer (ViT) model for versatile plant disease recognition, on multiple plant disease datasets. The method is first pre-trained in ImageNet with a selfsupervised loss function and with a supervised loss function in PlantCLEF2022, a large-scale dataset related to plants with 2,885,052 images and 80,000 classes. At last, one paper focused on appearance quality detection through detecting disease spots on pear fruits. Zhang et al. proposed an integrated framework combining instance segmentation, semantic segmentation and grading models, to assess the grading of the quality of the appearance of ‘Huangguan’ pear in a complex context. First, Mask R-CNN and Mask R-CNN with the introduction of the preprocessing module are used to segment ‘Huangguan’ pears from complex backgrounds; Second, DeepLabV3+, UNet and PSPNet are used to segment the ‘Huangguan’ pear spots to get the spots, and the ratio of the spot pixel area to the ‘Huangguan’ pear pixel area is calculated and classified into three grades; third, the grades of ‘Huangguan’ pear are obtained using ResNet50, VGG16 and MobileNetV3.

The other eight papers are dedicated to the study of insect pest detection and identification in this Research Topic. Most of papers focus on detecting multiple pests from complex background. To address the issues of pose-variant, serious overlap, dense distribution, and interclass similarity of agricultural pests, Jiao et al. proposed an end-to-end pest detection algorithm based on a deformable residual network to extract pest features and a global context aware module for obtaining region-of-interests of agricultural pests. Wang et al. addressed the issue of pest similarity in texture and scale, presented an ASP-Det to solve the texture-similarity problem and a Skip-Calibrated Convolution (SCC) module to balance the scale variation among the pest objects, and built a task-specific dataset named PestNet-AS that is collected and reannotated from PestNet dataset. Zhang et al. constructed a pest rotation detection (PRD21) using pest detection lamps in different natural environments, and performed a comparative study of image recognition through different target detection algorithms. The experimental results proved that rotation detection has a good effect on the detection and recognition rate of pests. Teng et al. proposed a robust pest detection network integrated with multiscale super-resolution (MSR) feature enhancement module to improve the detection performance of small-size, multi-scale, and high-similarity pests, and Soft-IoU (SI) mechanism to emphasize the position-based detection requirement by distinguishing the performance of different predictions with the same Intersection over Union (IoU). In addition, authors constructed a large-scale light-trap pest dataset (named LLPD-26), containing 26-class pests and 18,585 images with high-quality pest detection and classification annotations. Moreover, most methods required large-scale well-labeled pest datasets for their base-class training and novel-class fine-tuning, which hindered significantly the further promotion of deep convolutional neural network approaches in pest detection. Therefore, Wang et al. introducted a few-shot pest detection network to detect rarely collected pest species in natural scenarios. They presented a prior-knowledge-auxiliaried architecture for few-shot pest detection, built a hierarchical few-shot pest detection dataset in the wild in China over the past few years, and proposed a pest ontology relation module to combine insect taxonomy and inter-image similarity information.

Three papers focus on specific type of insect pests. Zhou et al. aimed at wheat spike detection and proposed a Transformer-based network named Multi-Window Swin Transformer (MWSwin Transformer) to use the ability of feature pyramid network to extract multi-scale features, integrated with self-attention mechanism by window strategy. They also proposed a Wheat Intersection over Union loss by incorporating the Euclidean distance, area overlapping, and aspect ratio. Furthermore, they constructed a wheat spike detection data set (WSD-2022) to evaluate the performance of the proposed methods. Liu et al. aimed at tomato pest detection and proposed a tomato pest identification algorithm based on an improved YOLOv4 fusing triplet attention mechanism (YOLOv4-TAM) with a focal loss function to address the issue of imbalances in the number of positive and negative sample images. They also used the K-means++ clustering algorithm to obtain a set of anchor boxes that correspond to the pest data set. Kalfas et al. aimed to detect Drosophila suzukii infestation in fruit orchards. They trained convolutional neural network (CNN) classifiers with frequency (power spectral density) and time-frequency (spectrogram) representations to distinguish D. suzukii insects from one of their closest relatives, Drosophila Melanogaster, based on their wingbeat patterns recorded by the optical sensor.

This Research Topic demonstrates several deep learning-based methods to address the issues of crop pest and disease detection occurred in real and complex world, and demonstrates how the use of deep learning methods can improve the understanding and detection of crop pests and diseases. Some research can also be used to decrease the loss of crop yield loss and increase crop production. We welcome everyone to explore the 16 research papers and improve their works in the future.

## Author contributions

PC drafted the manuscript. RW and PY checked the manuscript and suggested modifications. All authors contributed to the Editorial and approved the submitted version.

